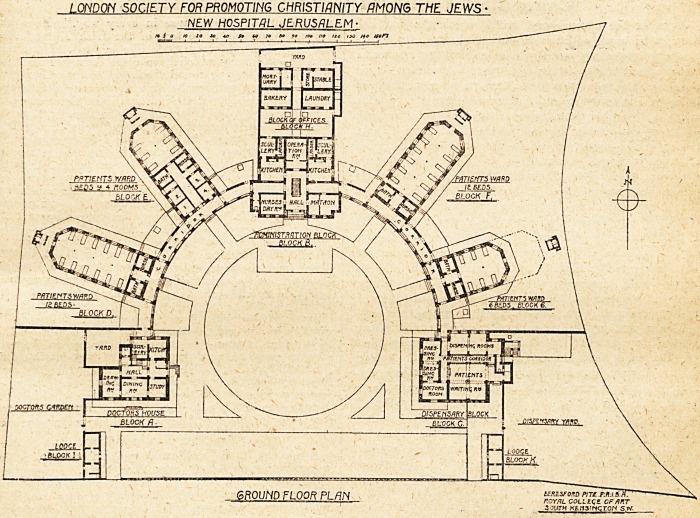# Church Missions Hospital, Jerusalem

**Published:** 1918-03-02

**Authors:** 


### HOSPITALS IN THE HOLY LAND.

### I.—Church Missions Hospital, Jerusalem.

The hospital of the Church Missions to the Jews at Jerusalem was built in 1894 upon a site on the Jaffa road, about a mile from the city gate. A study of local conditions was made previously, and the many hospitals belonging to various Churches and communities examined, there being a constant demand created amongst pilgrims for medical care. Most of these buildings, though large and heavily constructed with thick walls and vaults of the current modern Levant type, were most unsuitable for their purpose, and a few that had been designed with some regard for Western notions on the subject had proved failures owing to want of adaptation to climatic and other local conditions, *e.g.* absence of water supply or of drainage systems.

Among the main principles that emerged from this study upon the spot where the necessity of avoiding any direct exposure to the hot wind from the East, the raising of all floors above the earth line, and the provision of considerable air currents under as well as around the ward buildings, special thickness of walls, width and height of wards, and, as epidemic diseases not infrequently are manifested amongst the miscellaneous patients, provision for the complete isolation of any portion of the hospital. The only water supply is from heaven, therefore water storage for a year's rainfall in ventilated stone cisterns had to be provided, not below the floors of any rooms, as this had proved deleterious in a modern example. The site being upon rock, and sufficient earth and garden surface not being available, the employment of earth-closets was impossible; ventilated cesspits were provided for the detached latrines of each block. The slop-water was conveyed to a large central soak-away, but all gulleys adjacent to the building had to be covered to prevent mosquito breeding.

The native talent for simple stone buildings was relied upon with success. Problems of arching and doming, which prove costly in the West, are easily and economically handled by traditional skill in the East. The ward blocks are covered with stone and iron girder ceilings, and have, over these fire and heat-proof ceilings tile roofs on wooden rafters in order to avoid the constant pointing required to the usual flat stone roofs.

The scheme of the hospital plan is a horseshoe court with radiating pavilions, on either hand are the outpatients' and medical officers' residence blocks, in two storeys, and in centre administration block and nurses' residence. The wards are in single storeys, and are grouped for males and females on either side. Apart from finishings, but including the only simple joinery and sanitary fittings that were possible, the cost of this masonry worked out at the moderate sum of 6d. per foot cube. It was built by the employment of local labour without a contractor, and owing to careful supervision the proposed original expenditure was not materially exceeded. For more than twenty years the hospital has done a most beneficial work, which it is hoped may now be resumed.

### Some Practical Points.

The plan of this hospital is attractive from its simplicity and its symmetrical lay-out, which is suggestive of airiness and light. The absence of any water supply or system of sewerage must be a serious handicap to any sort of building, but must be specially a difficulty with hospital work. To discharge all the excreta and foul water into the bowels of the earth is a reversion to a barbarous and wasteful type; but with the local conditions no other course seems practicable. We imagine, however, that as regards the patients themselves, the conditions are pretty much those that obtain in a European hospital; and that such things as bedpans, urinals, and porringers are in just as much request as they would be here. Why, then, is there no provision for emptying and cleansing these necessary vessels? The very small detached sanitary annex to each ward block is apparently just large enough for one closet. The arrangement of the operation theatre does not seem altogether ideal. A room apparently about 16 ft. by 15 ft., approached through a lobby without direct light or ventilation, and with no accommodation for sterilising, or for the work necessary both in preparing for an operation and in clearing up afterwards, would not be regarded as sufficient accommodation in a cottage hospital. Neither is the position between the two kitchens and in the centre of the administration block altogether ideal for the purpose.

## Figures and Tables

**Figure f1:**